# Pathways to healing: balancing the tensions between harm reduction and cultural practices in a managed alcohol program for Indigenous adults in Calgary, Canada

**DOI:** 10.3389/fpubh.2026.1833954

**Published:** 2026-05-21

**Authors:** Angelina McLean, Katelyn Lucas, John Chief Moon, Ashley Prairie Chicken, Bernadette Pauly, Lara Nixon, Sarah Lamping, Jennifer Robinson, Meaghan Brown, Lauren Wan-Sai-Cheong, Lisa Zaretsky, Tiffany Morin, Katrina Milaney

**Affiliations:** 1 Department of Community Health Sciences, University of Calgary, Calgary, AB, Canada; 2 Aboriginal Standing Committee on Housing and Homelessness, Calgary, AB, Canada; 3 Department of Nursing, University of Victoria, Victoria, BC, Canada; 4 Canadian Institute for Substance Use Research, Victoria, BC, Canada; 5 Department of Family Medicine, University of Calgary, Calgary, AB, Canada; 6 Alpha House Society Calgary, Calgary, AB, Canada; 7 Department of Anthropology, University of Victoria, Victoria, BC, Canada; 8 Department of Psycholoy, University of Windsor, Windsor, ON, Canada

**Keywords:** community-based, harm reduction, housing, Indigenous ways of knowing, managed alcohol programs, Two-Eyed Seeing

## Abstract

**Objectives:**

This study aimed to examine the strengths and challenges of running a managed alcohol program for Indigenous adults in a non-Indigenous organization and posit areas to embed cultural practices as a necessary aspect for healing.

**Methods:**

Grounded in a Two-Eyed Seeing approach that weaved western and Indigenous ways of knowing and a community-based research design, this project included staff group and individual interviews followed by a Knowledge Keeper review. Cultural practices and ceremony guided the project from start to finish. Researchers used reflexive thematic analysis to co-create findings with Elders and community leaders.

**Results:**

Three key themes emerged from the staff interviews (1) success and strengths (2) challenges hindering full potential, and (3) recommendations for enhancement. Following data collection, Knowledge Keepers engaged in follow-up discussions and reflections on the project results to suggest a strength-based approach to embed cultural practices and cultural safety throughout the organization hosting the program.

**Conclusion:**

This study advocates for a movement beyond cultural programming toward fully integrating Indigenous knowledges into policies and practices for non-Indigenous organizations working with Indigenous adults. Reconciliation requires acknowledgement that culture and ceremony are necessary for healing. It also necessitates that Indigenous and non-Indigenous staff can actively engage in Indigenous ways of knowing to foster a culturally safe environment.

## Introduction

Managed alcohol programs (MAPs) were first popularized in Canada in the 1990s as a harm reduction approach to support individuals who were experiencing both homelessness and alcohol dependency (1). Unlike abstinence-based approaches, which may be unattainable for some, MAPs typically offer a measured amount of alcohol at regular intervals to help participants reduce their alcohol use and consume safer types of alcohol while disrupting cycles of homelessness and connecting people to care (2).

There are several MAPs operating in communities across Canada, many within mainstream organizations that work with residents from a variety of backgrounds, including Indigenous residents (1, 3, 4). Understanding the unique experiences of Indigenous residents in MAPs is particularly important because Indigenous peoples continue to be impacted by traumas related to the assimilation and silencing practices and policies of colonization (5). These traumas have caused high risks for unsafe substance use and the harms related to them including over-representation of Indigenous peoples in homelessness in Canadian cities (6). Importantly, there is a deep and long-standing tension between alcohol and Indigenous histories and traditions. Alcohol was introduced into Indigenous communities by colonizers as one method towards forced assimilation. It continues to be used “as a form of colonial knowledge” [(7) p. 58] to disempower Indigenous peoples through deeply entrenched and structural stereotypes that reinforce myths and misconceptions about Indigenous peoples.

In this study, we focus on staff experiences working with Indigenous residents in a non-Indigenous organization in Calgary, Alberta that supports Indigenous and non-Indigenous people. We bolster this with the wisdom of local Knowledge Keepers who contributed several ideas for knowledge mobilization including to enhance cultural practices in the MAP program. The purpose is to generate insights into what is needed to build culturally safe care within a non-Indigenous program. At the time of this study there was no MAP developed and operated exclusively by and with Indigenous peoples in Calgary, Canada.

### Background

Several studies on the effectiveness of MAP programs have emerged in Canada in the last several years. These studies have shown that while there are differences in the day-to-day operation of MAPs, their shared aim is to reduce the harmful effects of high alcohol consumption, including in some cases, non-beverage alcohol use (8, 9). Results from these studies show that those enrolled in MAPs consume less alcohol and have more consistent alcohol consumption patterns (8–10). MAPs have also been shown to reduce use of detox and emergency services as well as police encounters (9, 11). Zhao et al., (12) further demonstrated that being part of a MAP, reduced risk for mortality and morbidity including reduced hospitalizations. MAPs can also enable monitoring of other health conditions that may co-occur with alcohol intake such as liver function (10). In a study by Davis et al. (13) results showed that MAP residents reported improved access to regular and satisfactory healthcare.

Beyond health and system usage benefits, managed alcohol programs have also been shown to improve housing retention for formerly homeless individuals and enhance overall quality of life and wellbeing, increase agency and decision making, and provide a sense of home and family (14–16). Further, there is evidence to show that peer led, and peer run MAP programs help participants develop a sense of community (17).

The studies on the effectiveness of MAP programs noted above are bolstered by national guidance for MAP’s developed by The British Columbia Centre on Substance Use and the Canadian Institute for Substance Use Research (18). This guidance includes best practices for program models and sites, stakeholder consultation processes, necessary and ancillary services, space and staffing considerations as well as how to store and dispense alcohol to residents. Guidance for intake, assessment and eligibility considerations and individual case plans are also described. While the guidance is not specific to MAPs for Indigenous peoples, the authors discuss several important principles for MAPs including anti-racist practices, cultural safety and humility in addition to principles of harm reduction. The authors also emphasize the need for “Indigenous led services developed and operated through Indigenous leadership and with Indigenous clients” (p. 65).

### MAPs for Indigenous peoples

Ambrose Place in Edmonton, Alberta is one example of a MAP designed and led by and with Indigenous peoples. Ambrose Place is a supportive housing facility that provides wrap-around supports for trauma, mental health and substance use, primarily for Indigenous adults. Cultural practices are embedded within the MAP including access to Elders and ceremony onsite. In 2018 staff and researchers hosted sharing circles with residents to understand their experiences living at Ambrose Place. Residents shared that Ambrose felt like home and had created a family for them. They also talked about the importance of cultural support, its necessity for healing and a growing need for MAPs designed and led by and with Indigenous peoples (19).

In 2020, at the height of the COVID-19 pandemic, The Aboriginal Coalition to End Homelessness (ACEH) developed an Indigenous alcohol harm reduction residence program. This program is Indigenous led and informed by Elders from the three cultural families on Vancouver Island, Canada. Like Ambrose Place, the program is rooted in local Indigenous knowledges and includes access to Elders, ceremony and land-based healing within a culturally driven day-to-day program. A qualitative study of that program highlighted many positive results related to connection to culture for Indigenous residents (family members) (20). These included multiple and varied pathways to connecting to culture by offering a variety of opportunities for people to choose how and when to participate, based on what was best for them at that time. Family members in this study also talked about the differences they experienced between western abstinence based biomedical programs compared with Indigenous approaches. The latter being more collaborative and offering space to build trusting relationships and shared decision making (20).

Knowledge about MAP benefits, the overrepresentation of Indigenous peoples in homelessness, and the importance of weaving Indigenous ways of knowing into supports for healing for Indigenous individuals more broadly (20–22), necessitates an examination of MAPs within the context of the legacy impacts of colonization. We must also consider essential practice components for treatment systems that promote cultural safety, including practices that are free from racism and discrimination and provide opportunities for healing from the harms of unsafe alcohol use (23).

The research questions guiding this study were: 1. What are the strengths and challenges of running a MAP program for Indigenous adults in a non-Indigenous organization? 2. How can we enhance cultural safety in MAPs that support Indigenous residents but are not specifically led by Indigenous peoples?

## Methods

### Two-Eyed Seeing and ethical spaces

Results from this paper are from a larger four-year study examining MAP programs for Indigenous adults in both Calgary, Alberta and Vancouver Island, British Columbia, Canada. The lands of the peoples of Treaty 7 Territory and the unceded territory of the lək’ʷəŋən speaking and WSÁNEC peoples. Because the MAP site in Calgary, AB was a non-Indigenous organization working with Indigenous peoples, our approach focused on priorities identified during several Elder engagement sessions hosted by Calgary’s Aboriginal Standing Committee on Housing and Homelessness (ASCHH) that were in part, the impetus behind this study.

Calgary’s two-eyed approach (24) was supported by local Elders and included the creation of ethical spaces where both Western and Indigenous Ways of knowing, being, doing and connecting were woven together and applied in parallel (25). The project team from Calgary included Indigenous and non-Indigenous researchers, representatives from the ASCHH and Calgary’s Alpha House Society of Calgary (AHS). With the guidance of ASCHH, we embraced a collaborative, community-driven approach rooted in Indigenous epistemologies. We fostered respectful relationships with all partners, elevated voices equitably, while ensuring that Indigenous knowledges remained central throughout the study. Non-Indigenous partners on this project recognized and acknowledged their positions as historically privileged and so approached their roles as “followers, listeners and learners and deferred to Indigenous leadership in the research process” [(24), p. 14]. This approach was operationalized through our many ceremonies and land-based teaching opportunities and through regular in-person team meetings where in addition to research process discussions, team members reflected openly on their personal ideas and learnings, which created safe and ethical space for open dialogue and shared decision making. Our team meetings were hosted by ASCHH in a community space where we sat in a circle and started each meeting with a smudge and an opening blessing and we included time to critically reflect on what we were learning but also how we were working together and for what purposes (26), consistently centering the voices and experiences of Elders and Indigenous community leaders.

Indigenous methodologies that honour relational and experiential ways of knowing, community connection and engagement guided every stage of the project. This included time spent building relationships and trust between the researchers, program staff, Elders and Knowledge Keepers before data collection; audio recording of interviews and transcription and in-person, oral discussions to share interview content (27). Analysis was done collaboratively through several stages of thematic analysis as well as sharing circles and ceremony. Examples include research team co-creation of themes, presentation of early themes to our Elders circle, and acknowledgment of our results through a traditional Blackfoot pipe ceremony. Authorship of manuscripts and presentations are inclusive of these groups. We launched our project in 2021 with a series of land-based teachings facilitated by five Elders representing the Kainai Nation, Siksika Nation and Bearspaw First Nation at the creation site of the Blackfoot peoples in Alberta and our findings were acknowledged with Elders with a tipi raising and Tea Dance ceremony in 2024.

In order to respect Indigenous data sovereignty, all data in this study was shared amongst the research team. This means, all team members had equal rights to collect, own and utilize the data (28). This study was approved by the University of Calgary Conjoint Health Ethics Research Board, REB20-0391 while adhering to the principles of Ownership, Control, Access and Possession (OCAP) and CARE principles for Indigenous data governance (29, 30).

### Setting

The Calgary community organization (AHS), the site of the MAP for this study, launched their MAP in 2015 to provide formerly homeless Indigenous and non-Indigenous residents with harm reduction supports in their housing buildings. At that time, the program was referred to as the Beer Budgeting Program (BBP). In 2017 the program shifted to offering a MAP program that included liquor and wine, to enable more people to access the program. Staffing at the five participating buildings include 1–2 case workers per shift, who support MAP participants 24 h a day, 7 days a week with a team lead present and team managers available with oversight for several buildings. The case workers assist with transportation to health appointments, facilitating referrals and connections to community services, and helping residents manage their alcohol use throughout the day. The case workers store alcohol in a secure office and track residents’ consumption throughout the day. The team managers oversee the MAP and meet with the residents monthly to discuss substance budgeting. Residents participating in the MAP volunteer to access the program and pay for their own alcohol based on a monthly budget they co-create with staff.

## Data collection

### Staff interviews

A purposive sampling strategy was used to recruit staff for a first round of interviews as we wanted to hear from people with direct knowledge of the MAP program or the cultural supports that were offered at the organization. We applied non-discriminative snowball sampling for a second round to broaden our reach and leverage staff connections which was helpful for our phased approach to data collection, the exploratory nature of our study and ensuring we reached saturation (31, 46).

Data was collected from staff in two ways. First, in late 2021 and early 2022 we led two group interviews with MAP staff to understand their experiences working in the program including strengths and areas for growth. Following preliminary analysis of results, our research team decided to follow up with one-on-one interviews in April of 2023 in order to facilitate deeper and richer discussions. Staff participants held a variety of roles in the organization including case managers, team leaders and program managers. Length of time working at the organization varied from several weeks to several years. This diversity of perspectives ensured we could include a variety of experiences, and we believe added richness to the data (32). These interviews were conducted online during staff breaks to ensure minimal disruption to their work schedules. In total, nine staff attended the group interviews (six in one and three in another) and 17 participated in one-on-one interviews. Six of the same staff participated in both rounds. Because we used the same interview guides for each, we included data from both sources in this paper. Group interview quotes are coded as G1 or 2 and individuals are coded as I1,2,3, etc.

### Analysis

Three researchers independently coded the group transcripts, and two researchers independently coded the individual interview transcripts. We explored themes and patterns in our data and used NVivo 14 software to organize the codes. The analysis followed Braun and Clarke’s six-phase process: (1) familiarization (2) initial coding (3) theme development (4) reviewing (5) refining, and (6) identifying higher-order themes (33). Researchers developed a codebook, which was referenced throughout the analysis with weekly debriefing amongst the coders (34). When there was less than 100% agreement, the researchers engaged in discussions to resolve differences and reach a consensus. Several standard checks were conducted to ensure the quality of the data (35). To ensure the trustworthiness of the data, another researcher reviewed the final themes and codes (36). Credibility checks were undertaken at all stages in the analysis (37). The researchers presented the preliminary findings to ASCHH, to the Elders and AHS all of whom provided oral recommendations and contextual insights, significantly enriching the analysis. Their feedback was documented and subsequently incorporated into the coding framework and thematic structure.

### Knowledge keeper review

Following analysis of group and individual interviews, a theme was emerging related to tensions between harm reduction and participation in ceremony. The ASCHH co-chair suggested two Knowledge Keepers engage in small group discussions with staff once again, to better understand the cultural practices currently happening in the organization and to bring forward their recommendations for enhanced cultural support from their worldviews and cultural teachings. The Knowledge Keepers, with an Indigenous student researcher hosted discussions with 12 of the staff who participated in the interviews. These discussions were not considered part of formal data collection, as they were conducted specifically to meet our obligations to the Elders and AHS regarding mobilizing the findings into tangible solutions. The Knowledge Keepers met with the ASCHH co-chair (KL) and the lead academic (KM) to report their findings orally. Orally reporting the findings honored and respected Indigenous tradition and protocol (38). Following the oral report, KL and KM met to talk through what the Knowledge Keepers shared. We then conducted a manual thematic analysis independently, then met to discuss synergies and areas of difference. Where there were differences, as co-chair of ASCHH, KL contributions were prioritized. We then co-created a written report and presented the themes back to the Knowledge Keepers to ensure we appropriately honored their words. After their approval, the report was shared with the leadership team of AHS including recommendations, for their consideration. The distinction in the purpose of these discussions (not formal data collection but related to mobilization of knowledges), was clarified and approved by the University of Calgary Conjoint Health Research Ethics Board and is aligned with our Two-Eyed Seeing approach, honoring Indigenous knowledges.

## Results

Three themes were identified by staff as being central to: (1) successes and strengths of the program; (2) challenges hindering the program’s full potential, and (3) recommendations for program enhancement. Following presentation of the three themes, recommendations from the Knowledge Keeper ideas have been summarized specifically related to mobilizing the results into practices to enhance cultural (re)connection as an embedded aspect of the MAP, as well as throughout the organization.

### THEME 1: key successes and strengths of the program

This theme includes three subthemes (1) reduced and healthier alcohol use (2) improved safety and less isolation (3) residents have choice and autonomy.

#### Reduced and healthier alcohol use

Staff emphasized that the MAP significantly helped decrease withdrawal symptoms and use of non-beverage alcohol among MAP residents. Staff also reported reduced alcohol use overall. This was shared in almost every interview.

*“I also think just from a harm reduction perspective, people are living with addiction, they don't need to suffer…I think it's just basic dignity to wake up and not have to feel awful and sick. Alcohol withdrawal can kill you, so I think [the MAP] is fantastic.” I10*

*“A lot of the time, you know, you hear that people utilize a lot of alcohol or perhaps non beverage alcohol. And they may indicate that they want to make a change so that conversation can come very organically … but there is definitely less non beverage alcohol for sure.” G1*

*“Binge drinking isn't as prevalent. And it also just like helps with the behaviors that come with intoxication. Like everything's just like a little bit more controlled and spread out” G1*

#### Improved safety and less isolation

Staff reported that residents in the MAP developed a daily routine and did not leave the building as often. This meant they could avoid potentially unsafe situations in community where they might be exposed to unsafe substances or even violence.

*“I think it's also nice how it almost establishes a routine for the clients of like, no, this is something that I can go to the office and get, and then like, it doesn't put them in situations or be vulnerable in the community… So if we can minimize the risk that clients are at for vulnerability, I think that's also a side to it that is important to note.” G2*

Part of the routine was coming to the office creating opportunities for interactions, relationship building and supporting access to other services.

*I think it also kind of prevents the isolation as well that most clients would go through. Right. If they're just sitting at home by themselves, here, when they have to come down for their alcohol, they're not isolating as much. They're not shaming themselves as much… So yeah, I think it's very beneficial for their mental health.” I8*

*“Sometimes there'd be clients. You wouldn't see them like throughout the day, you know, you'd have to kind of go out of your way to check on them. And then if they're on the program, suddenly you're seeing them every hour and then sometimes that can help to get them other services like medical services sometimes or seeing a nurse…” G1*

Ultimately, the MAP contributed to reducing loneliness and isolation.

#### Residents having choice and autonomy

A final subtheme under MAP program strengths was that residents have a choice over whether they wish to be part of the program, it is not mandatory or tied to their housing. Once people choose to be part of the MAP, they make decisions with staff that include their alcohol budget, their choice of alcohol and any other support they may want to access.

*“So if they're interested… we'll have that discussion with them on what it's about, it also, helps with budgeting as well. So they kind of meet with us, and kind of decide how much money that they wanna contribute towards the MAP program… We would purchase the items and then the client has access to the beer once every hour or so just to manage, their intake.” G1*

The program is personalized to each resident based on their needs and wishes, which staff believed helps people stay in the program.

*“There's no one way of doing it with each client… We're pretty flexible with how we can make it work for them.” I8*

*“We have a conversation of asking them, would they like to have assistance with their alcohol management? And then we have a further discussion about the amount that they would like to contribute to it… then going from that, we have a conversation of what it would mean for them in terms of what would be like their product of choice… and so it's sustainable." I4*

This subtheme demonstrates the value that staff place on including residents in decision making about their care.

### THEME 2: areas for growth

This theme noted some challenges in the program’s delivery that staff wanted to address. We frame these as areas for growth including (1) alcohol budget limitations (2) Occupational distress for staff (2) tensions between alcohol use and culture.

#### Alcohol budget limitations

Staff shared that some participants did not have enough money to pay for their alcohol for the full month, which caused a lot of stress for the residents and the staff and the need to find alternative sources of funding for the alcohol. At the time of this study, funding for alcohol fell exclusively to residents, all of whom were living on very low incomes.

*“They go through [money] really fast within the first few days of the month… So come the end of the month, once they've exhausted all their [alcohol] and they don't have money, yeah, they're at high risk of withdrawal or maybe drinking non-bev.” I8*

*“I mean, in terms of changing things, obviously I think it would be the funding for it. That's the main thing… but some of them have an advantage over others where they can last throughout the whole month, where others might not.” I11*

*“So it's finding ways… to support the residents who may only have $150 for the entire month to get a few bottles of alcohol or cases of beer that's supposed to be longstanding. And sometimes you see people get frustrated with that and give up on the program… but how do we go about supporting that when they don't have the finance piece.” I12*

Lack of funding for alcohol was a commonly identified issue. This led to concerns about residents being at risk of withdrawal, drinking non-beverage or pursuing alternative substances to fill the gap.

#### Occupational distress for staff

This subtheme highlighted that staff were working under very stressful circumstances. This included managing alcohol intake for residents and related to the subtheme above, this was particularly difficult when residents ran out of alcohol.

*“It leads to a lot of staff trauma, just because people will get violent and things like that if they're told no. At the end of the day, we keep saying no, because it's just what we have to do.” I5*

*“I feel like it's very hard managing someone else's alcohol, just in general…Or being so strict on time can be tough on both clients and staff. And then also, it takes a lot out of your workday.” I12*

*“Because I think one of the slight barriers… is that when they do get used to every day they come down and get their beer, when they do run out, it's our fault. Because we're the people that hand out the beer, so we should have the beer.” I9*

Staff also highlighted the effects of COVID-19 pandemic which exacerbated their stress.

*“Prior to the pandemic, we would've had a variety of sessions… and then clients were assisted by like transport and whatnot …I think honestly, the pandemic caused a lot of delay in us being able to do that for our clients, which was very upsetting.” G2*

#### Tensions between alcohol and culture

Staff reported that many of the Indigenous residents wanted to participate in cultural practices and ceremonies, but they acknowledged that many traditions require sobriety to participate.

*“I know we've been racking our brains trying to explore ways to get that going… we do have access to things like pipe circles and drumming and different pieces within the community, but in that as well, it's usually a sobriety requirement. So that is a barrier in a lot of ways”. G1*

*“You see people really wanting to engage, but they can't, or don't know how. Also, there's so much loss of culture that I think some people don't necessarily know where to start…G2*

Residents often felt embarrassment or shame when they could not participate because of their alcohol use.

*“I think with substance use, there's often a lot of shame in engaging in culture and ceremony, just because that wasn't part of their culture…So a lot of people when they're using won't engage because they do feel a lot of negative feelings surrounding that. So, often they just don't because there's so much trauma that it's choosing the cultural piece or choosing the outlet to kind of forget the trauma. So usually that outlet wins”. G1*

Staff also reported difficulties finding Elders who represented a person’s cultural background.

*If we do have someone that’s a cultural support, like an Elder, it’s not from their background. So they may not be as inclined to engage…I’ve heard that a lot. Like he’s Metis…That is not my culture”. G1*

In other examples, staff talked about the importance of finding ways to support people to be sober long enough to participate.

*“One of our clients wanted her room to be smudged, but she realized because she had been drinking, you're not able to partake in those kinds of cultural activities. I know the sweats that the detox clients go to… the guy who runs it, he's very open since it's his sweat…can we support housing clients to stay sober enough to go?” G2*

### THEME 3: staff ideas for program enhancement

Staff proposed recommendations that fell into two main subthemes: (1) changes to address funding issues; and (2) social connections, including (re)connection to culture for residents.

#### Changes to address funding issues

Staff suggestions included a backup fund in case a resident does run out of their monthly alcohol supply.

*“I would have to say maybe for a period of time, some extra funding for the people who are going to go hard into withdrawal…" I4*

Staff also suggested revisiting resident budgets partway through the month to try and avoid issues later.

*“So coming up with a plan, so that they don't go through withdrawal. So it doesn't become dangerous for them to suddenly be out of [alcohol], have a cushion for when they run out. So that we make sure that they're still safe.” I8*

*“So maybe even revisiting it halfway through the month and saying, "I don't know. You've only got two beers left, so what would we like to do? How can we do this to better suit you so that we've got something here until the beginning of next month?" I1*

*“I think it's maybe more planning, more structuring. And just being proactive… So that in times when the client has exhausted all their [alcohol], there's a way for them to still access, so that they don't go into withdrawal. I think that's the biggest thing for them and for us.” I9*

#### Social connections including (re)connection to culture

Staff felt that increasing social events could help build relationships.

*“Yeah. Even once a month to start, and once [social events] gain momentum, maybe twice a month. But I think that's really important, and it would be good for them too, just to socialize, to get out of their units and meet other people.” I14*

*“Other supports that they could get would be maybe having social meetings with others that are on the [MAP], just for them to interact. I find clients, they do really have a huge impact on each other… “Okay, everyone on [MAP], let's come and discuss, how are you experiencing this? How are you liking this?”… And also, if other people in the building see, "Oh, it's actually working for others," they might be motivated to be like, "Hey, you know what? It's actually working for my friend ABC. Let me try it and see…” I13*

Staff also suggested offering more traditional practices as well as opportunities to begin a conversation with an Elder, even if it was not participation in a formal ceremony.

*“I would love to see beading, drumming, cooking…just sitting with Elders and talking…Maybe they can’t smudge or go to a sweat but having a chance to talk about it with an Elder… encouraging each other, and the staff just being there for crafts, baking, cooking, and all that…” G1*

*“It's just hard to get them outta the building really. Like you kind of have to bring these things to them, for them to be more likely to participate …” G2*

### Knowledge keeper recommendations

Results from the staff interviews revealed many strengths and opportunities for growth. Because this MAP is run by a non-Indigenous organization that supports many Indigenous residents, and because the issue of access to cultural supports emerged quite strongly, ASCHH suggested we invite two Knowledge Keepers with an understanding of homelessness and substance use to talk with staff further and provide ideas to build cultural practices organizationally. Because this step of the project was specific to Indigenous led ideas to respond to the themes in the data, we present Knowledge Keepers’ feedback outside of the formal themes. We confirmed with our Indigenous community partners and our institutional ethics review board that this was an acceptable process related to the mobilization of research findings rather than research themes.

The suggestions that follow were offered by the Knowledge Keepers as ways to begin to rebuild cultural practices following the COVID-19 pandemic and to design strategies to incorporate access to traditional practices in safe and comprehensive ways throughout the organization. These suggestions are meant to help embed culture and ceremony as organizational *practices* rather than standalone programs only available for the MAP residents.

#### Enhance cultural identity

*“You can’t teach culture or lived experience in university” Knowledge Keeper*

The Knowledge Keepers advised that consideration should be given to embedding culture and ceremony in many layers across the organization. This includes hiring and staff orientation practices as well as access to Elders and ceremony. These practices should be extended to all staff and residents, Indigenous and non-Indigenous. This could be operationalized by hiring a “*cultural consultant*” to develop a comprehensive organizational strategy with policies and procedures for recruitment and staff training as well as direct guidance for residents wanting to (re)connect with their cultural teachings and ceremonies. The Knowledge Keepers also suggested recruiting “*cultural liaisons*” or people who embody strong traditional values who could provide a cultural support system for staff and help bridge connections for collaborations with Indigenous communities and services. Additionally, they recommended employing peer support workers, creating guidelines for supporting them and revising hiring criteria to value lived experience as equal to academic credentials.

#### Deepen Indigenous awareness training

“*There is a difference between culture and ceremony… there is a difference between living it and growing into it and a difference between learning about it later” Knowledge Keeper*

The Knowledge Keepers reflected that staff who are not Indigenous may not fully understand the pathways to trauma for Indigenous peoples nor be able to fully relate to their healing journeys. Learning about alcohol use from a traditional perspective in parallel with western perspectives would allow staff to see the issue of alcohol more effectively and help with understanding how trauma is linked to alcohol use. Knowledge Keepers felt this was just as important as understanding harm reduction approaches.

The Knowledge Keepers recommended expanding Indigenous Awareness Training beyond standard accreditation minimums. This could involve organizing cultural retreats and land-based teachings and encouraging staff to participate in ceremonies alongside program participants. Experiential learning activities, such as the Blanket Exercise, which is a teaching ceremony about settlers as they took away lands, languages and traditions was one suggestion. This was suggested as a way to enhance understanding of historical trauma and subsequent homelessness and alcohol issues.

#### Strengthen support systems for staff

*“Regular debriefs are not enough…staff stress levels are high, and they should have access to an Elder once a week to smudge and to talk.” Knowledge Keeper*

The Knowledge Keepers emphasized the importance of hiring as many Indigenous staff as possible and building internal support systems to foster ongoing rapport, connection and healing for staff so that occupational distress could be reduced. Daily smudging was suggested, led by someone who is qualified to assist with the teachings and to be there as a guide. These supports should be available to both Indigenous and non-Indigenous staff.

## Discussion

Results from our study highlight important strengths of the MAP program we studied. Program strengths included healthier alcohol use, improved safety and less isolation for the residents as well as improved choice and autonomy which meant the program could be personalized and adapted based on each resident’s wishes and needs. Important areas for growth included addressing limitations with how budgets for alcohol purchases were managed, difficulties for staff in managing residents’ alcohol use which caused some distress and very importantly, deeply embedded tensions between alcohol use and participation in cultural practices and ceremonies. Staff proposed several ideas including diverse funding models and opportunities for social and cultural (re)connection. The inclusion of ideas from the Knowledge Keepers is an incredibly important contribution from our project. Their suggestions included enhancing the cultural identity of the organization as well as deepened Indigenous awareness training and support systems for all staff, Indigenous and non-Indigenous.

Many of our results are consistent with previous literature, including improved housing stability and reduced and healthier alcohol use overall (2, 8–10). Similarly, improved autonomy for residents, largely because of the harm reduction and client-centered approach that helped manage withdrawals and increase safety (15). Many staff members were able to engage better with residents who tended to stay in the building more. This reduced risks and vulnerability for residents if they were out in community and exposed to unsafe situations. Participating in the MAP also helped create a sense of safety and community.

Program challenges were exacerbated by the COVID-19 pandemic which limited opportunities for social activities as well as on-site and community access to cultural and ceremonial activities. Staff also talked about tensions between alcohol use and culture which limit opportunities to participate in ceremonies and engage with Elders.

It is important to note that since this data was collected, the community organization created a partnership with a brewery to provide beer to residents who run out before the end of the month. There are MAPs in Canada that do onsite brewing which helps reduce the costs associated with purchasing alcohol from a retail outlet. The Brew Co-op, is one example in a peer run and peer led MAP in Vancouver (17). There are examples from programs in other jurisdictions who have created alternative funding models to also help address this gap. For example, a joint funding model where residents and organizations, health authorities or government funders share the costs (39, 40).

The findings related specifically to challenges and recommendations for Indigenous residents adds value to broaden the knowledge and scholarship about MAP programs. While staff acknowledged the complexities and tensions between alcohol use and ceremony, it was suggested that finding Elders and community leaders who would be comfortable coming to a building where there is active substance use could be an important first step. Staff suggestions in our study for expanded supports for safety and community are in line with Indigenous principles of healing that highlight wholistic ways of growth that extend beyond the biomedical model of addiction and focus on the growth of the person (41, 42). In another study where all the MAP participants self-identified as Indigenous, the importance for residents to have a home, not just a house, a place where they could develop community, and relationships, was an example of ways to support people to begin their journey within a MAP (15).

A specific strength of our project was the inclusion of the Knowledge Keepers’ review and subsequent recommendations which bolster the staff identified challenges and suggestions for growth. This aspect of our study adds to the scholarly discourse and ‘best practice’ approaches particularly for non-Indigenous organizations who work with Indigenous peoples.

The first recommendation from the Knowledge Keepers was to enhance cultural identity throughout the organization, embedding cultural practices, not just programs. They suggested working with Indigenous consultants, liaisons and adding lived expert peer support workers to enhance staff hiring and orientation practices as well as embed varied opportunities for access to Elders and ceremony.

The second recommendation was to deepen Indigenous awareness training, so it is more meaningful for all the organization’s employees, especially those working in the MAP. This included land-based teachings, cultural experiences, and exercises from Elders and Knowledge Keepers, side-by-side with residents. A final recommendation was to hire as many Indigenous staff as possible while ensuring all staff had access to cultural supports including for occupational distress.

Our Two-Eyed Seeing approach enabled practical and tangible suggestions for program improvement from the staff who work in programs every day and from cultural leaders who brought their teachings and traditional ways of knowing to suggest ideas for embedded and consistent cultural practices, not just programming. This is a particular strength and contribution from this project.

This study has other strengths. First, all aspects were guided by Indigenous Elders and ceremony. This enhanced the trustworthiness and meaningfulness of the results and recommendations. Second, the engagement of key community players such as the MAP organization and ASCHH. ASCHH ensured this research was rooted in Indigenous and community-based perspectives and expertise. The community partner operating the MAP was also involved in all aspects of this study which helps with the integration of the study results and recommendations. As noted in the discussion above, the organization has already taken steps to address the challenges and implement suggestions to enhance growth.

Third, this study included two rounds of interviews with staff followed by discussions with two Knowledge Keepers, this allowed us to explore staff perspectives and experiences with more nuance and depth, from western and Indigenous paradigms. Furthermore, the research team included both Indigenous and Non-Indigenous interviewers, one of whom had frontline experience working with the community partner. This insider perspective may have helped staff feel more comfortable sharing their experiences. These results can help make important contributions, for example, expanding on the guidelines from the BC Centre for Substance Use and Canadian Institute for Substance Use Research, for developing and operating MAP program to include tangible and practical ways to embed cultural safety, humility and anti-racist practices (2023).

## Limitations

This study has several limitations. First, the findings presented in this paper do not include the experiences of residents. While staff experiences were important to understand how to enhance programming, hearing from the residents themselves is a critical component to understanding how the program itself promotes individual healing. Results from resident interviews are discussed in a separate manuscript currently in development, but presenting the data in these ways limits what can be said holistically about the MAP program. Second, this study took place in the wake of the COVID-19 pandemic. Group and one-on-one staff interviews took place online. While some researchers suggest that online interviews can be just as helpful as in-person interviews (43), there can still be some barriers to facilitating connection and trust building (44). Third, interviews were conducted during staff breaks to minimize work disruptions. This may have limited responses if staff felt time-constrained or unable to focus on the interview if many other work tasks were distracting them. However, two rounds of interviews with staff meant we were able to achieve data saturation whereby no new information was emerging by the end of round two (45).

Lastly, it is important to recognize that these study findings are situated in the context of the MAP we examined in Calgary, Alberta, Treaty 7 territory. While some experiences and findings may be similar in different contexts, they should be interpreted with caution when considering them in different settings and within different Indigenous communities, leaders and cultural traditions.

## Conclusion

Results from this study are grounded in a Two-Eyed Seeing approach which allowed for deep and authentic partnerships and community engagement, adding meaning and relevance to the results and recommendations. Examining strengths and challenges from the staff perspectives highlighted several important benefits for MAP residents as well as suggestions for program enhancements. Contributions from the Knowledge Keepers led to several ideas for enhanced cultural practices and cultural safety, specifically by suggesting ways to embed changes in practice and policy across multiple levels of the organization, not just within the MAP.

This paper makes two important contributions, first, we believe our methodological approach can serve as an example for other academic researchers on how to challenge oppressive power structures and work with Indigenous community leaders as learners and listeners rather than as Eurocentric academic ‘experts’. We also believe the ideas from the Knowledge Keepers, will be helpful for other organizations who support Indigenous peoples but may not be led specifically by Indigenous peoples. Our results could applied in other communities within their own contexts and with their own local partners.

There is significant diversity and heterogeneity among Indigenous cultures across Canada, and it is essential to note that findings should be localized to each community’s needs and traditions. We also acknowledge that the perspectives offered in this paper are not inclusive of the residents in the MAP program, and consideration of lived experiences would further enhance the suggestions posited here.

## Data Availability

The datasets presented in this article are not readily available as per Indigenous data sovereignty practices. Requests to access the datasets should be directed to katrina.milaney@ucalgary.ca.

## References

[1] Pauly B Vallance K Wettlaufer A Chow C Brown R Evans J . Community managed alcohol programs in Canada. Drug Alcohol Rev. (2018) 37:S132–9. doi: 10.1111/dar.12681 29573059

[2] Pauly B Brown M Evans J Gray E Schiff R Ivsins A . There is a place’: impacts of managed alcohol programs. Harm Reduct J. (2019) 16:70. doi: 10.1186/s12954-019-0332-4, 31842903 PMC6916004

[3] Motta-Ochoa R Incio-Serra N Poliquin H MacDonald S Huỳnh C Côté P . “A place to be safe, feel at home and get better”: including the experiential knowledge of potential users in the design of the first wet service in Montreal, Canada. Harm Reduct J. (2022) 19:34. doi: 10.1186/s12954-022-00616-6, 35382814 PMC8985343

[4] Moledina A Myran D Patel R Simmons JG. Practical considerations for residential-managed alcohol programs: lessons from Ottawa Inner City health. Harm Reduct J. (2026) 23:25. doi: 10.1186/s12954-025-01360-3, 41645188 PMC12879482

[5] Rodriguez C Henderson R Lucas K Bristowe S Bromley J Chief Moon M . Challenging structural racism and violence in policy and practice for indigenous families experiencing homelessness. Int Indig Policy J. (2024) 15:1–16. doi: 10.18584/iipj.2024.15.3.16334

[6] Smye V Browne AJ Josewski V Keith B Mussell W. Social suffering. Int J Environ Res Public Health. (2023) 20:3288. doi: 10.3390/ijerph20043288, 36833982 PMC9958899

[7] Quintero G. Making the Indian: colonial knowledge, alcohol, and native Americans. Am Indian Cult Res J. (2001) 25:57–71. doi: 10.17953/aicr.25.4.d7703373656686m4

[8] Stockwell T Zhao J Pauly B Chow C Vallance K Wettlaufer A . Trajectories of alcohol use. Alcohol Alcohol. (2021) 56:651–9. doi: 10.1093/alcalc/agaa134, 33418568

[9] Vallance K Stockwell T Pauly B Chow C Gray E Krysowaty B . Do managed alcohol programs change patterns? Harm Reduct J. (2016) 13:13. doi: 10.1186/s12954-016-0103-4, 27156792 PMC4860767

[10] Stockwell T Pauly B(B) Chow C Erickson RA Krysowaty B Roemer A . Does managing consumption reduce harm? Drug Alcohol Rev. (2018) 37:S159–66. doi: 10.1111/dar.12618 29027283

[11] Podymow T Turnbull J Coyle D Yetisir E Wells G. Shelter-based managed alcohol administration. CMAJ. (2006) 174:45–9. doi: 10.1503/cmaj.1041350, 16389236 PMC1319345

[12] Zhao J Stockwell T Pauly B Wettlaufer A Chow C. Participation in Canadian managed alcohol programs. Alcohol Alcohol. (2022) 57:246–60. doi: 10.1093/alcalc/agab078, 34999760

[13] Davis A Pauly B Vallance K. Access to healthcare among managed alcohol program participants. Drug Alcohol Depend. (2025) 272:109961. doi: 10.1016/j.drugpo.2025.104950, 40829298

[14] Evans J Semogas D Smalley JG Lohfeld L. ‘This place has given me a reason to care’: understanding managed alcohol programs as enabling places in Canada. Health Place. (2015) 33:118–24. doi: 10.1016/j.healthplace.2015.02.011, 25817940

[15] Pauly B Gray E Perkin K Chow C Vallance K Krysowaty B . Finding safety: a pilot study. Harm Reduct J. (2016) 13: 1–11. doi: 10.1186/s12954-016-0102-5, 27156564 PMC4860766

[16] Smith-Bernardin SM Suen LW Barr-Walker J Cuervo IA Handley MA. Scoping review of managed alcohol programs. Harm Reduct J. (2022) 19:82. doi: 10.1186/s12954-022-00646-0, 35879719 PMC9311344

[17] Pauly B King V Smith A Tranquilli-Doherty S Wishart M Vallance K . Breaking the cycle of survival drinking. Drugs Educ Prev Policy. (2020) 28:72–180 doi: 10.1080/09687637.2020.1764500

[18] British Columbia Centre on Substance Use, and Canadian Institute for Substance Use Research Managed Alcohol Programs—Canadian Operational Guidance Victoria SURE (2023). Available online at: https://www.substanceuse.ca/managed-alcohol-programs-canadian-operational-guidance (Accessed March 18, 2026).

[19] Dumais N. Pascal N. Cunningham C. tawāw pe-Apik • Poohsapoot … come and sit and be at home (2018). Available online at: https://www.uvic.ca/research/centres/cisur/assets/docs/report-cmaps-ambrose-place.pdf (Accessed March 18, 2026).

[20] Brown M Hunt-Jinnouchi F Robinson J Clark N Mushquash C Milaney K . ‘Give me the reigns of taking Care of Myself with a home’: healing environments in an indigenous-led alcohol harm reduction program. Harm Reduct J. (2024) 21:177. doi: 10.1186/s12954-024-01090-y, 39327559 PMC11426064

[21] Asamoah GD Khakpour M Carr T Groot G. Exploring indigenous traditional healing programs in Canada, Australia, and New Zealand: a scoping review. Explore (NY). (2023) 19:14–25. doi: 10.1016/j.explore.2022.06.004, 35768321

[22] King M Smith A Gracey M. Indigenous health part 2: the underlying causes of the health gap. Lancet. (2009) 374:76–85. doi: 10.1016/S0140-6736(09)60827-8, 19577696

[23] Urbanoski K Pauly B Inglis D Cameron F Haddad T Phillips J . Defining culturally safe primary care. BMC Health Serv Res. (2020) 20:1060. doi: 10.1186/s12913-020-05915-x, 33228650 PMC7685616

[24] Wright AL Gabel C Ballantyne M Jack SM Wahoush O. Using two-eyed seeing in research. Int J Qual Methods. (2019) 18. doi: 10.1177/1609406919869695

[25] Colbourne R Moroz P Hall C Lendsay K Anderson RB. Indigenous works and two-eyed seeing: mapping the case for indigenous-led research. Qual Res Organ Manag. (2020) 15:68–86. doi: 10.1108/QROM-04-2019-1754

[26] Martin DH. Two-eyed seeing: a framework for understanding indigenous and non-indigenous approaches to indigenous Health Research. Can J Nurs Res. (2012) 44:20–43. 22894005

[27] Wilson S. Research Is Ceremony: Indigenous Research Methods. Black Point: Fernwood Publishing (2008).

[28] Paul M. Looking to the future: indigenous data sovereignty and policy in Canada. Pathfinder. (2023) 4:54–67. doi: 10.29173/pathfinder71

[29] First Nations Information Governance Centre. Ownership, Control, Access and Possession (OCAP): the path to first nations information governance. (2014). Available online at: https://www.rd-alliance.org/wpcontent/uploads/2024/03/CARE20Principles20for20Indigenous20Data20Governance_OnePagers_FINAL20Sept2006202019.pdf. (Accessed March 18, 2026).

[30] Global Indigenous Data Alliance. CARE principles for indigenous data governance. (2019). Available online at: https://achh.ca/wpcontent/uploads/2018/07/OCAP_FNIGC.pdf. (Accessed March 18, 2026).

[31] Naderifar M Goli H Ghaljaie F. Snowball sampling: a purposeful method of sampling in qualitative research. Strides Dev Med Educ. (2017) 14:1–6. doi: 10.5812/sdme.67670

[32] Campbell S Greenwood M Prior S Shearer T Walkem K Young S . Purposive sampling: complex or simple? Research case examples. J Res Nurs. (2020) 25:652–61. doi: 10.1177/1744987120927206, 34394687 PMC7932468

[33] Braun V Clarke V. Thematic Analysis: A Practical Guide. London: SAGE Publications (2022).

[34] Janesick VJ. "Peer Debriefing". In: The Blackwell Encyclopedia of Sociology. Hoboken: Wiley (2015)

[35] Yadav D. Criteria for good qualitative research. Asia Pac Educ Res. (2022) 31:679–89. doi: 10.1007/s40299-021-00619-0

[36] Nowell L Norris J White D Moules N. Thematic analysis: striving to meet the trustworthiness criteria. Int J Qual Methods. (2017) 16. doi: 10.1177/1609406917733847

[37] Barker C Pistrang N. Quality criteria under methodological pluralism: implications for conducting and evaluating research. Am J Community Psychol. (2005) 35:201–12. doi: 10.1007/s10464-005-3398-y, 15909795

[38] Christensen J. Telling stories: exploring research storytelling as a meaningful approach to knowledge mobilization. Can Geogr. (2012) 56:231–42. doi: 10.1111/j.1541-0064.2012.00417.x

[39] Motta-Ochoa R Marsan S Incio-Serra N Audéoud E-R Talbot A Temgoua MM . Barriers and facilitators to implementing a first managed alcohol program in Montreal, Canada. Drugs Educ Prev Policy. (2025) 32:532–43. doi: 10.1080/09687637.2025.2470129

[40] Pauly B Reist D Belle-Isle L Schactman C. Housing and harm reduction. Int J Drug Policy. (2013) 24:284–90. doi: 10.1016/j.drugpo.2013.03.008, 23623720

[41] Absolon K. Indigenous wholistic theory: a knowledge set for practice. First Peoples Child Fam Rev. (2019) 14:22–42. doi: 10.7202/1071285ar

[42] McCabe GH. The healing path: a culture- and community-derived indigenous therapy model. Psychother Theory Res Pract Train. (2007) 44:148–60. doi: 10.1037/0033-3204.44.2.148, 22122207

[43] de Villiers C Farooq MB Molinari M. Qualitative research interviews using online video technology—challenges and opportunities. Meditari Account Res. (2022) 30:1764–82. doi: 10.1108/MEDAR-03-2021-1252

[44] Archibald MM Ambagtsheer RC Casey MG Lawless M. Using zoom videoconferencing for qualitative data collection: perceptions and experiences of researchers and participants. Int J Qual Methods. (2019) 18:1609406919874596. doi: 10.1177/1609406919874596

[45] Hancock ME Amankwaa L Revell MA Mueller D. Focus group data saturation: a new approach to data analysis. Qual Rep. (2016) 21:2124–30. doi: 10.46743/2160-3715/2016.2330

[46] Korstjens I Moser A. Series: Practical Guidance to Qualitative Research. Part 2: Context, Research Questions and Designs. European Journal of General Practice (2017) 23:274–79. 29185826 10.1080/13814788.2017.1375090 PMC8816399

